# The Effect of Peppermint and Thyme Oils on Stabilizing the Fatty Acid Profile of Sunflower Oil

**DOI:** 10.3390/molecules29020292

**Published:** 2024-01-05

**Authors:** Radosław Kowalski, Grażyna Kowalska, Przemysław Mitura, Rafał Rowiński, Urszula Pankiewicz, Joanna Hawlena

**Affiliations:** 1 Department of Analysis and Food Quality Assessment, University of Life Sciences in Lublin, 8 Skromna Str., 20-704 Lublin, Poland; urszula.pankiewicz@up.lublin.pl; 2 Department of Tourism and Recreation, University of Life Sciences in Lublin, 15 Akademicka Str., 20-950 Lublin, Poland; grazyna.kowalska@up.lublin.pl (G.K.); badaniaawf@wp.pl (R.R.); joanna.hawlena@up.lublin.pl (J.H.); 3 Department of Urology and Oncological Urology, Medical University of Lublin, 8 Jaczewskiego Str., 20-954 Lublin, Poland; przemyslaw.mitura@umlub.pl

**Keywords:** sunflower oil, fatty acid composition, peppermint and thyme oils, oil storage

## Abstract

Presently, there is an increasing shift towards the utilization of natural antioxidants and compounds with protective attributes for fatty acids in order to replace synthetic counterparts that may pose health risks. This transition aligns with the growing emphasis on promoting healthy and organic food choices. Essential oils stand out in this context due to scientific validations of their antioxidant properties. There are few published research results concerning changes in the fatty acid composition in model systems with the addition of essential oils. This study aims to investigate the impact of incorporating peppermint and thyme oils on inhibiting changes in the fatty acid profile of sunflower oil stored at both room temperature with exposure to daylight and in a thermostat set at 40 °C. The experimental procedure involved the addition of peppermint and thyme oils, along with butylated hydroxyanisole (BHA), to batches of sunflower oil. The samples were then stored for 11 months. The study observed a detrimental influence of storage conditions on the quantitative changes in the fatty acid profile of the sunflower oil. The addition of BHA stabilized the content of linoleic acid in the sunflower oil (approximately 53 g/100 g of linoleic acid compared to approximately 58 g/100 g in the control sample). Meanwhile, the model system of sunflower oil with the addition of peppermint and thyme oils (40 °C) exhibited a statistically significant decrease in the concentration of linoleic acid to approximately 8 g/100 g after eleven months of thermostating. Similar trends to those observed for linoleic acid were noted for the total fatty acid content in the sunflower oil. Notably, the efficacy of the selected substances in inhibiting adverse transformations in fats was contingent upon their concentration and the storage temperature.

## 1. Introduction

**Figure 1:**
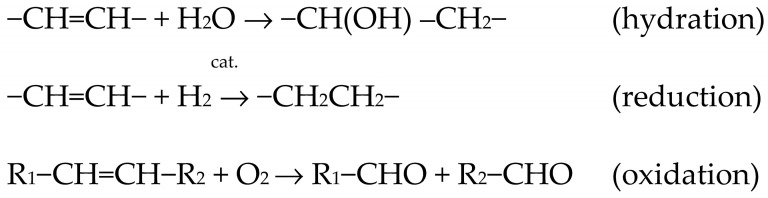
Characteristic reactions of unsaturated fatty acids [3,4,5].

Lipids primarily serve as reserve materials for storing energy, and their heat of combustion is nearly twice as high as that of carbohydrates and proteins. Fats are the most calorically dense components of food [1]. Dietary fats include commonly used plant oils, such as olive oil, rapeseed oil, and sunflower oil, as well as animal fats like butter, pork lard, and beef tallow [2]. The current trend in nutrition indicates a growing interest among consumers and producers in vegetable oils, most of which are a source of essential polyunsaturated fatty acids (PUFAs). Polyunsaturated fatty acids are characterized by the presence of double bonds between carbon atoms in the carbon chain. Fatty acids that contain multiple double bonds (2, 3, 4, 5, or 6) are called polyunsaturated or polyenoic acids. Unsaturated fatty acids undergo reactions such as hydration, reduction, and oxidation (Figure 1).

Polyunsaturated fatty acids undergo undesired transformations during storage, thermal processing (culinary processes), and various technological processes. Undesirable changes in fats include hydrolysis, oxidation (peroxides and hydroperoxides, which subsequently give rise to aldehydes and ketones), polymerization, and cyclization. The composition of such fats includes compounds that significantly reduce the nutritional value of fats [6]. One of the simplest solutions is to add substances (including antioxidants) to fats that will inhibit adverse changes.

Essential oils have been known for centuries as agents for preserving food (due to the antioxidant properties of essential oils) or flavoring ingredients [7,8,9]. Previous studies have shown that rosemary and marjoram oil macerates exhibited greater stability in the composition of fatty acids compared to the original rapeseed oil [10,11]. Moreover, adding essential oils (rosemary, marjoram) to rapeseed oil in some model systems helped stabilize the oil composition [6,10,11]. Essential oils, as very complex natural mixtures of volatile compounds, can also accelerate undesired reactions, as they may contain components initiating such reactions. Essential oils also undergo spontaneous oxidation [12].

The essential oils obtained from plants of the Lamiaceae family are especially interesting [13]. In this regard, peppermint and common thyme should be mentioned, as these species belong to valuable medicinal and culinary plants containing essential oils with a wide range of applications [14]. Species from the *Mentha* genus are among the oldest medicinal and aromatic plants in the world, but the most useful (due to a high content of oil and a wonderful fragrance composition) is peppermint (*Mentha* × *piperita* L.) [15]. Peppermint oil (*Menthae piperitae oleum*) obtained by steam distillation is approved for medicinal use and, like peppermint, has broad applications in the industry [16]. The characteristic scent of peppermint oil is attributed to the compound menthol, a monoterpenoid alcohol, which can constitute up to 60% of the essential oil [17]. This alcohol has bactericidal properties, but an excess of menthol can have negative effects on the nervous system. Peppermint oil possesses spasmolytic, carminative, choleretic, and diuretic properties, as well as sedative, disinfectant, anti-inflammatory, flavoring, analgesic, soothing, diaphoretic, digestive-stimulating, and menstruation-inducing effects [18].

Common thyme (*Thymus vulgaris* L.) has been used for various purposes (medicinal and culinary) for a long time. Common thyme herb contains 3.5 to 5.4% essential oil, with up to 50% of the thyme oil components being phenolic derivatives. Thymol is the main component, reaching up to 76%, and carvacrol up to approximately 84%. The composition of individual thyme oils depends largely on cultivation and geographical factors. The main components of the oil obtained from thyme grown in southeastern Poland are thymol, γ-terpinene, *p*-cymene, and carvacrol [19,20]. Thyme oils from the Netherlands (65.5%) and Estonia (75.7%) are characterized by the highest thymol content, while oils from plants in Greece are dominated by carvacrol (83.5%), and thyme oil from Armenia contains only 17.0% thymol [21]. Due to the high content of thymol and carvacrol, thyme oil can be used as an antioxidant instead of BHT [22]. Carvacrol and thymol have shown similar antioxidant activities compared with BHT and BHA in linoleic acid emulsion tests at different concentrations [23]. Thyme oil, characterized by strong expectorant properties, can increase the number of leukocytes in the human body, contributing to the improvement of the body’s defense functions. Thyme is widely used worldwide as a spice. Thyme herb is characterized by a strong aroma and is an excellent addition to various dishes.

Unfortunately, there is a limited amount of research evaluating changes in the fatty acid composition of plant oils enriched with natural additives with potential stabilizing properties, such as essential oils.

Considering that plant oils in the human diet are a significant source of essential unsaturated fatty acids, and taking into account the possibility of unfavorable transformations of these fatty acids, research aimed at determining the impact on these transformations of components that often serve as natural additives to oils is justified. Such additives capable of improving the aromatic qualities of plant oils include essential oils, providing extensive possibilities for composing products in the category of flavored oils. In the presented study, the influence of adding peppermint oil (*Mentha* × *piperita* L.) and common thyme oil (*Thymus vulgaris* L.) on inhibiting changes in the fatty acid profile of sunflower oil stored at both room temperature with exposure to daylight and in a thermostat set at 40 °C is explored.

## 2. Results and Discussion

The dominant fatty acids in the fatty acid profile of the sunflower oil used in the experiment were unsaturated fatty acids, primarily di-unsaturated linoleic acid (18:2) and monounsaturated oleic acid (18:1), as evidenced by the analyses. This clearly indicates that the oil is an important source of these acids (Table 1).

**Figure 2:**
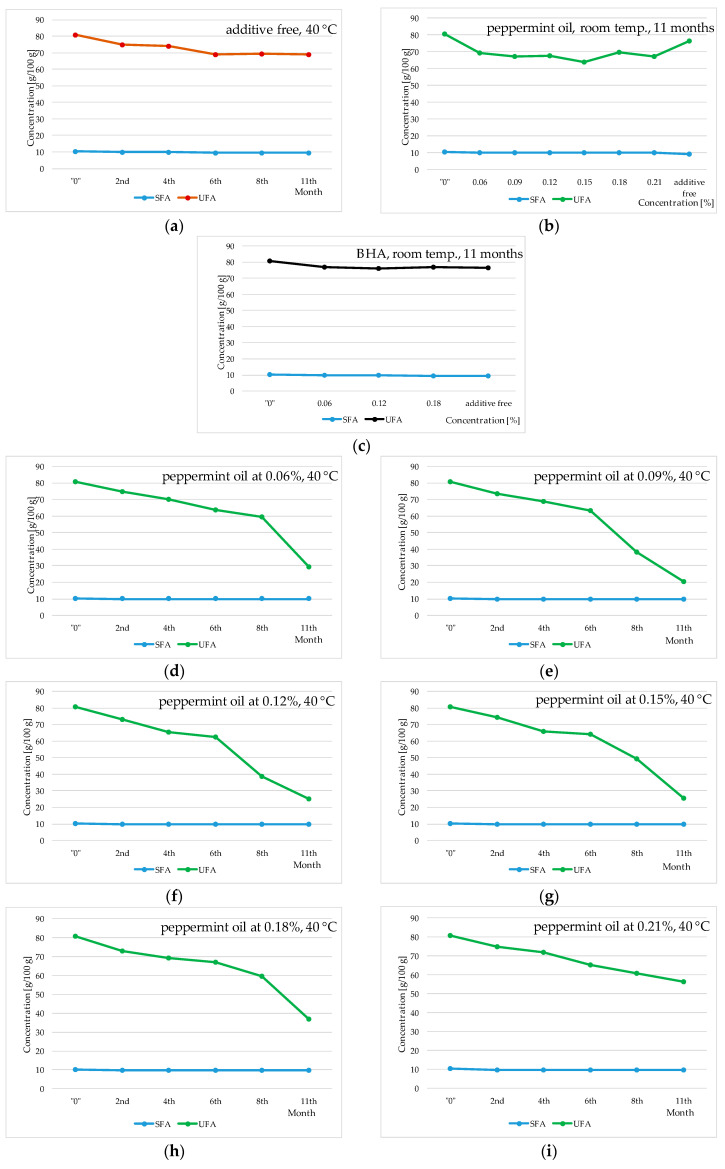
Changes in saturated and unsaturated fatty acids (SFAs and UFAs) in sunflower oil stored at room temperature (RT) and thermostated at 40 °C with addition of peppermint oil and BHA; “0”—initial oil samples. (**a**) Sunflower oil with no additions, stored at 40 °C for 2, 4, 6, 8 and 11 months. (**b**) Sunflower oil with addition of peppermint oil at 0.06%, 0.09%, 0.12%, 0.15%, 0.18% and 0.21% stored at room temperature for 11 months. (**c**) Sunflower oil with addition of BHA at 0.06%, 0.12% and 0.18% stored at room temperature for 11 months. (**d**) Sunflower oil with addition of peppermint oil at 0.06% stored at 40 °C for 2, 4, 6, 8 and 11 months. (**e**) Sunflower oil with addition of peppermint oil at 0.09% stored at 40 °C for 2, 4, 6, 8 and 11 months. (**f**) Sunflower oil with addition of peppermint oil at 0.12% stored at 40 °C for 2, 4, 6, 8 and 11 months. (**g**) Sunflower oil with addition of peppermint oil at 0.15% stored at 40 °C for 2, 4, 6, 8 and 11 months. (**h**) Sunflower oil with addition of peppermint oil at 0.18% stored at 40 °C for 2, 4, 6, 8 and 11 months. (**i**) Sunflower oil with addition of peppermint oil at 0.21% stored at 40 °C for 2, 4, 6, 8 and 11 months.

**Figure 3:**
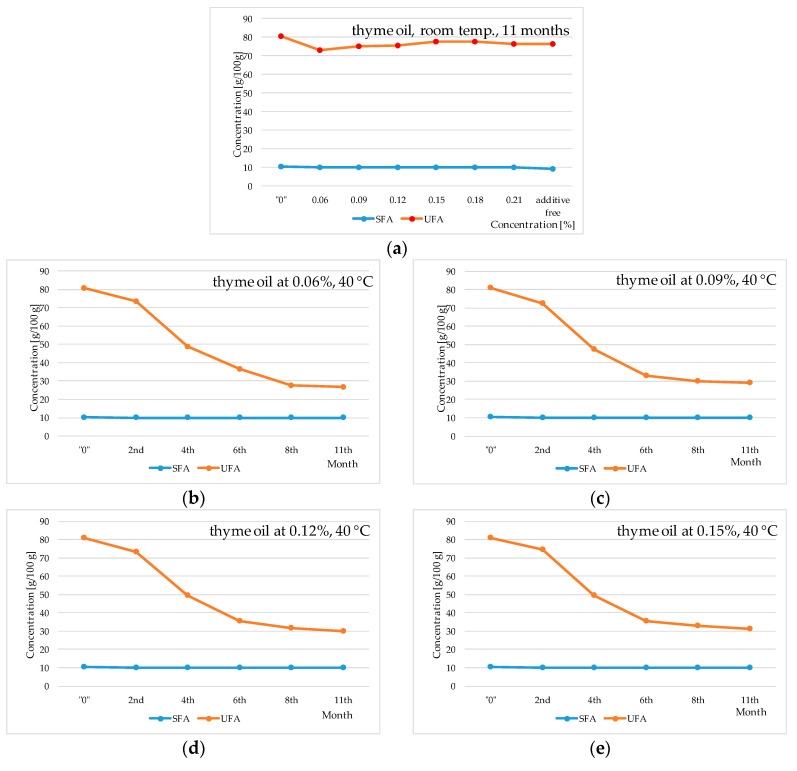

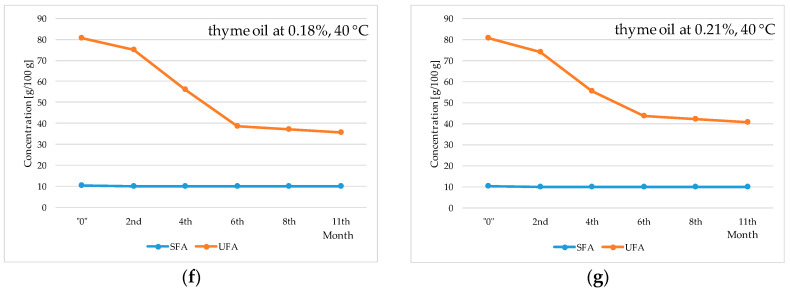
Changes in saturated and unsaturated fatty acids (SFAs and UFAs) in sunflower oil stored at room temperature (RT) and thermostated at 40 °C with addition of thyme oil; “0”—initial oil samples. (**a**) Sunflower oil with addition of thyme oil at 0.06%, 0.09%, 0.12%, 0.15%, 0.18% and 0.21% stored at room temperature for 11 months. (**b**) Sunflower oil with addition of thyme oil at 0.06% stored at 40 °C for 2, 4, 6, 8 and 11 months. (**c**) Sunflower oil with addition of thyme oil at 0.09% stored at 40 °C for 2, 4, 6, 8 and 11 months. (**d**) Sunflower oil with addition of thyme oil at 0.12% stored at 40 °C for 2, 4, 6, 8 and 11 months. (**e**) Sunflower oil with addition of thyme oil at 0.15% stored at 40 °C for 2, 4, 6, 8 and 11 months. (**f**) Sunflower oil with addition of thyme oil at 0.18% stored at 40 °C for 2, 4, 6, 8 and 11 months. (**g**) Sunflower oil with addition of thyme oil at 0.21% stored at 40 °C for 2, 4, 6, 8 and 11 months.

**Figure 4:**
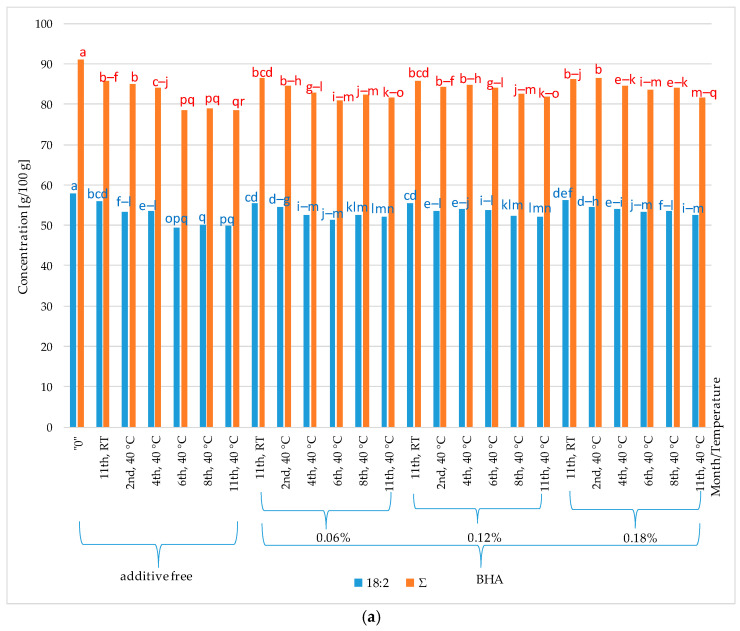

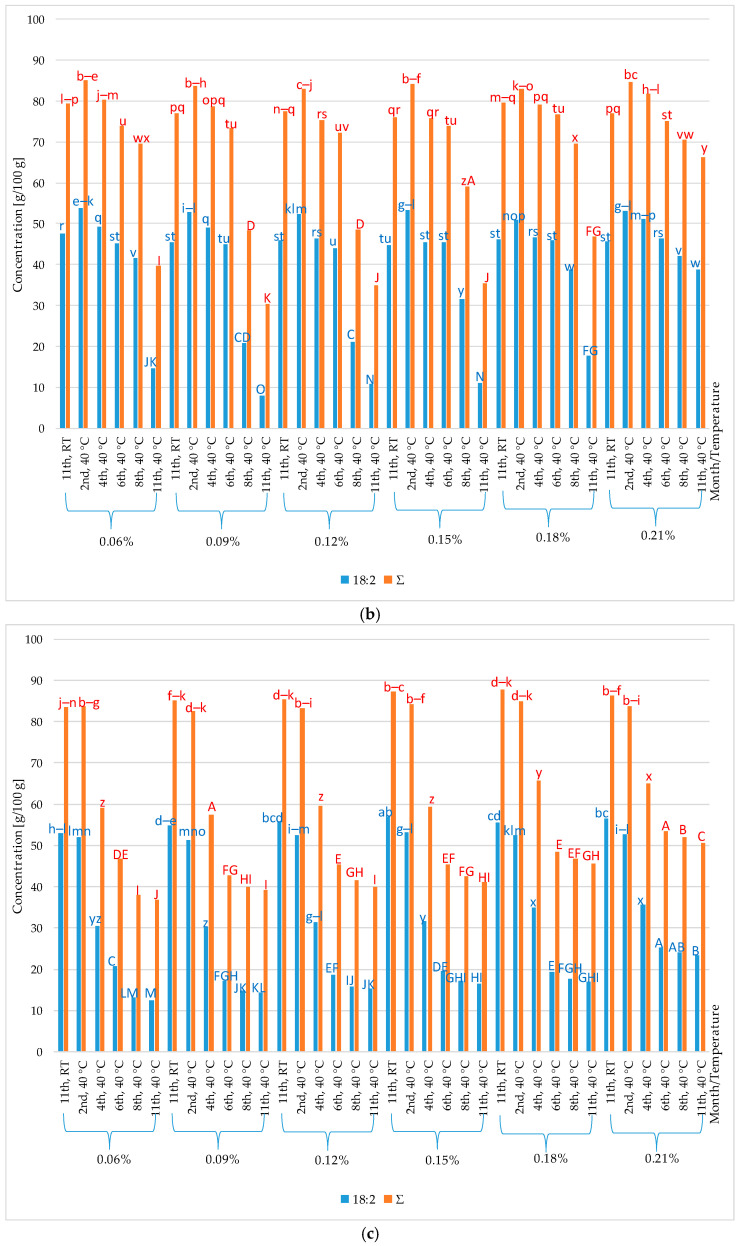
Content of linoleic acids and total concentration of fatty acids in sunflower oil with addition of peppermint and thyme oils and BHA; “0”—initial oil samples; a, b, c, d … A, B, C, D …—values designated with the same letters do not significantly differ at 5% error (Duncan’s test). (**a**) Sunflower oil with no additions and with addition of BHA at 0.06%, 0.12% and 0.18% stored at 40 °C for 2, 4, 6, 8 and 11 months, and stored at room temperature (RT) for 11 months. (**b**) Sunflower oil with addition of peppermint oil at 0.06%, 0.09%, 0.12%, 0.15%, 0.18% and 0.21% stored at 40 °C for 2, 4, 6, 8 and 11 months, and stored at room temperature (RT) for 11 months. (**c**) Sunflower oil with addition of thyme oil at 0.06%, 0.09%, 0.12%, 0.15%, 0.18% and 0.21% stored at 40 °C for 2, 4, 6, 8 and 11 months, and stored at room temperature (RT) for 11 months.

The conducted experiment revealed that an elevated storage temperature (40 °C) induces a reduction in the concentration of fatty acids, as depicted in Figure 2, Figure 3 and Figure 4. Notably, the concentration of linoleic acid, along with the total fatty acid content, exhibited a proportional decline with the duration of oil storage at 40 °C. In contrast, when the oil was stored for 11 months at room temperature, the decrease in fatty acid content was significantly lower. Specifically, for linoleic acid it was approximately 2.1 g/100 g instead of around 8.0 g/100 g (at 40 °C), and for the total sum of fatty acids it was about 5.3 g/100 g instead of approximately 12.5 g/100 g (at 40 °C). These findings align with analogous trends observed in prior studies [6].

Comparing the results for control samples stored under the same conditions with the results for samples with the addition of peppermint and thyme oils, as well as butylated hydroxyanisole (BHA), it can be stated that the applied essential oils exhibit inferior properties compared to the synthetic additive BHA. BHA positively influences the maintenance of a constant level of linoleic acid (approximately 53 g/100 g–58 g/100 g). However, the addition of peppermint and thyme oils to sunflower oil (40 °C) is associated with statistically significant quantitative changes in fatty acid content in the investigated experimental systems over the storage time (approximately 58 g/100 g in the initial sunflower oil and approximately 8 g/100 g in the oil after eleven months of thermostating). Similar trends to those observed for linoleic acid were noticed for the total fatty acid content in sunflower oil. The addition of BHA exhibited a positive stabilizing effect, influencing the maintenance of a constant quantitative level for the total fatty acids in the sunflower oil of approximately 91 g/100 g and approximately 78 g/100 g, respectively. In contrast, the addition of peppermint and thyme essential oils showed statistically significant reductions in the total fatty acid content, i.e., at room temperature, approximately 76 g/100 g–80 g/100 g (peppermint oil) and 83 g/100 g–88 g/100 g (thyme oil), and at a temperature of 40 °C the content decreased to approximately 30 g/100 g (peppermint oil) and approximately 37 g/100 g (thyme oil). Therefore, it can be inferred that thermostating at 40 °C accelerates unfavorable changes occurring in the fatty acid composition of both the initial oil and the oil with the addition of peppermint and thyme oils. Kowalski [24,25,26] and Kowalski et al. [6] also confirm that heating vegetable oils leads to an absolute decrease in the concentration of fatty acids, especially linoleic acid. This is due to the fact that elevated temperature increases the reaction rate occurring in fats. Milczarek and Osek [27] demonstrated in their research that the oxidation rate of lipids for rapeseed products was slower at low temperatures. Significantly higher quantitative changes were observed in samples thermostated at 40 °C compared to those stored at room temperature. Relating these results to the data obtained for samples with the addition of peppermint and thyme oils, it can be stated that the examined essential oils exhibit catalytic action that accelerates changes occurring in fats, resulting in a decrease in the concentration of polyunsaturated fatty acids.

**Figure 5:**
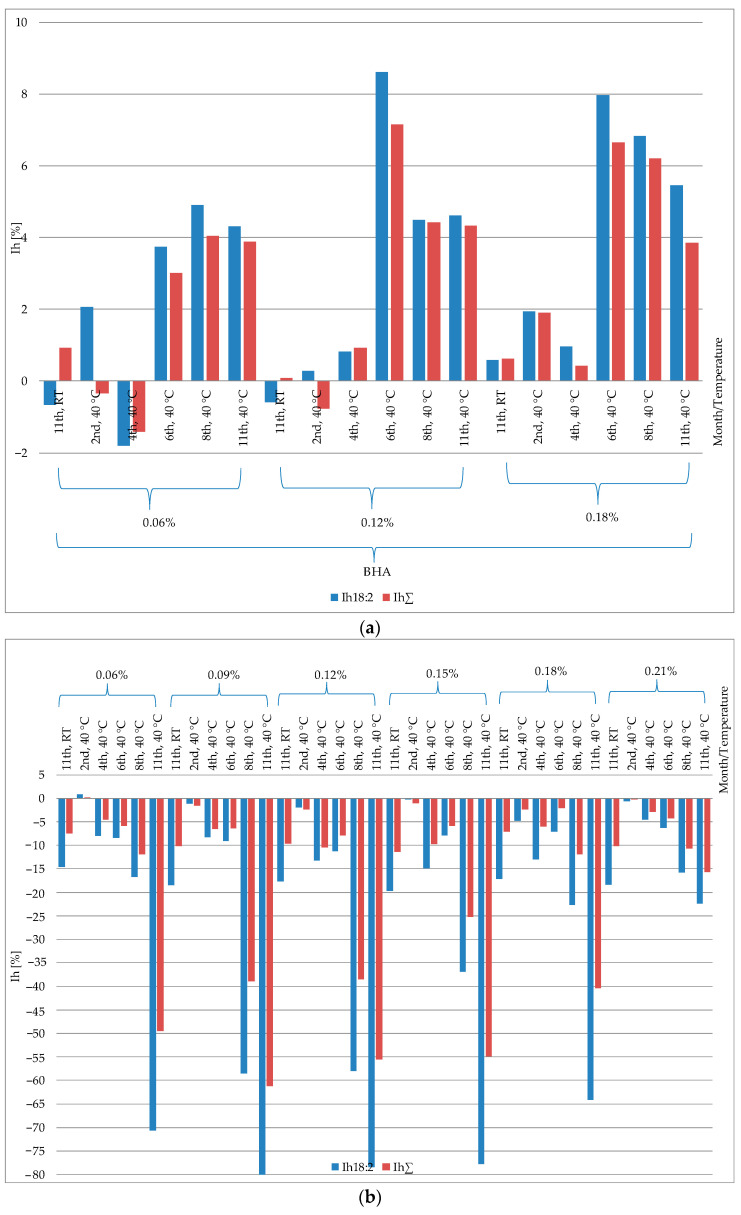

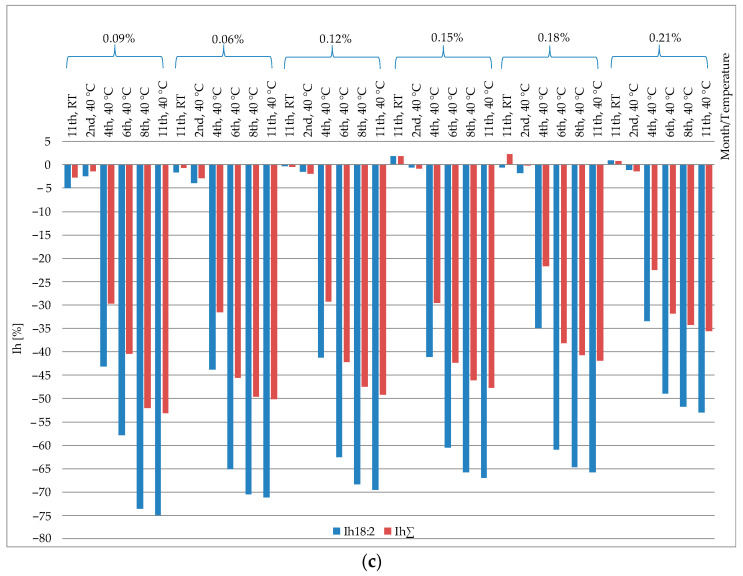
Effectiveness of peppermint and thyme oils and BHA at inhibition of quantitative changes in linoleic acid (I_h18:2_) and total fatty acid content (I_hΣ_) in sunflower oil. (**a**) Sunflower oil with addition of BHA at 0.06%, 0.12% and 0.18% stored at 40 °C for 2, 4, 6, 8 and 11 months, and stored at room temperature (RT) for 11 months. (**b**) Sunflower oil with addition of peppermint oil at 0.06%, 0.09%, 0.12%, 0.15%, 0.18% and 0.21% stored at 40 °C for 2, 4, 6, 8 and 11 months, and stored at room temperature (RT) for 11 months. (**c**) Sunflower oil with addition of thyme oil at 0.06%, 0.09%, 0.12%, 0.15%, 0.18% and 0.21% stored at 40 °C for 2, 4, 6, 8 and 11 months, and stored at room temperature (RT) for 11 months.

For linoleic acid (18:2), the highest inhibition values (I_h18:2_) were observed in the oil systems stored in the thermostat (40 °C, 6 months). I_h18:2_ amounted to 8.61% for the samples with the addition of BHA in concentrations of 0.12%, and I_h18:2_ was 7.97% for concentrations of 0.18% BHA. In the case of oil variants stored at 40 °C (2 months), the inhibition value obtained for the sample with the addition of peppermint oil in a concentration of 0.06% was I_h18:2_ = 0.82% (Figure 5). In the case of peppermint oil, it was the only positive value indicating the inhibition of changes in the concentration of linoleic acid. On the other hand, the addition of thyme oil was associated with the observation of inhibiting changes in the concentration of linoleic acid in sunflower oil stored at room temperature for 11 months in concentration systems of 0.15%—I_h18:2_ = 1.88% and 0.21%—I_h18:2_ = 0.97%. Regarding the inhibition coefficient for quantitative changes in the sum of fatty acids, the highest values were observed for the addition of BHA: I_hΣ_ = 0.08% (BHA 0.12% room temperature) and I_hΣ_ = 7.15% (BHA 0.12%, 40 °C, 6 months).

With the specific experimental variants, the applied BHA exhibited no discernible inhibitory effect on unfavorable alterations in the fatty acid profile of the analyzed oil. Conversely, a decrease in fatty acid content was noted, resulting in negative inhibition values: I_h18:2_ = −0.66% (BHA 0.06% at room temperature), I_h18:2_ = −0.59%(BHA 0.12% at room temperature), I_h18:2_ = −1.81%, I_hΣ_ = −1.42% (BHA 0.06% at 40 °C for 4 months), I_hΣ_ = −0.34% (BHA 0.06% at 40 °C for 2 months), and I_hΣ_ = −0.77% (BHA 0.12% at 40 °C for 2 months). Following the sample preparation procedure, fat undergoes initial saponification (alkaline hydrolysis) and, subsequently, the fatty acids are converted into methyl esters [28]. Fatty acids that, under the experimental conditions, transformed into stable compounds such as polymers of fatty acids, oxygen derivatives of fatty acids (oxidized fatty acids), or cyclic compounds, are impervious to reactions performed during the fatty acid assay. Consequently, they cannot be assayed in the methyl esters group, as observed in the quantitative composition of individual fatty acids with a declining trend in concentrations over the storage period of the samples [6].

Besbes et al. [29] illustrated that palm oil subjected to a temperature of 100 °C for 48 h exhibited markedly higher viscosity compared to unheated oil, a consequence attributed to the formation of carbon–carbon and carbon–oxygen–carbon-type bridges between fatty acids [30,31]. Simultaneously, the observed quantitative shifts in the fatty acid content of the analyzed fats were undeniably instigated by the oxidation of these compounds through exposure to atmospheric oxygen, resulting in the creation of oxide forms of fatty acids. The oxidation process of fatty acids intensifies at elevated temperatures and correlates with the qualitative composition of fatty acids in the analyzed fats. Fats with significantly dominant polyunsaturated acids in their profile, such as linseed oil, sunflower oil or soybean oil, are more susceptible to oxidation. Consequently, fats rich in polyunsaturated acids should not be stored at room temperature, a common occurrence on store shelves. Furthermore, alterations in the quality of analyzed fats may be induced by substances coexisting with the fats, such as those found in butter, potentially serving as nutrients for bacteria and triggering fermentation processes, evident through rancidity and heightened oxidation. Elevated temperatures act as a facilitator for the oxidation process of fatty acids, leading to the production of undesirable compounds and thereby causing fats to lose their anticipated properties and become devoid of valued antioxidants [24].

In a previous study, it was demonstrated that the addition of marjoram oil to rapeseed oil in certain experimental setups resulted in inhibiting changes in the fatty acid composition with an efficacy similar to the synthetic antioxidant BHA [6].

While the topic of enhancing the stability of fats with natural additives, including essential oils, is addressed in scientific research, unfortunately there is a lack of sufficient studies evaluating quantitative changes occurring in the fatty acid profiles of the examined fat systems. Halilović et al. [32] investigated the stability of flaxseed oil with the addition of essential oils from garlic, thyme, and oregano, showing that the addition of thyme oil affected a decrease in the stability of the tested system with flaxseed oil compared to other essential oils. The results of other published studies are not unequivocal and are difficult to compare. Micić et al. [33] examined the oxidation induction time (OIT) of sunflower oil with different proportions of added rosemary essential oil at a temperature of 140 °C, finding that an increase in the concentration of these additives resulted in a significant reduction in OIT, indicating a decrease in the stability of sunflower oil. Unfortunately, the described study did not include an assessment of the fatty acid composition. In another study involving the addition of essential oils (clove, patchouli, and citronella) to biodiesel from palm oil (olein), the authors demonstrate that the additives contributed to the improvement of biodiesel stability [34]. However, this study also did not examine the profile of methyl esters, and the assessment utilized indirect methods such as acid number, peroxide number, DPPH antioxidant activity test, Rancimat test, viscosity, and Composite Performance Index analysis. The cited authors emphasize that increase in temperature is a determining factor in oxidative changes, reducing the stability of the examined model systems.

In the current experiment, however, the addition of peppermint and common thyme oils was employed. Peppermint oil (Table 2) was dominated by the following components: menthone (31.26%), menthol (25.71%), and menthyl acetate (10.50%), confirming previous literature reports. Góra and Lis [35] reported that approximately 300 components of peppermint essential oil have been identified, with menthol (from 24.1% in France to 59.4% in Russia) and menthone (from 4.5% in France to 32.1% in Poland) being the main ones. In the essential oil of *Mentha piperita*, components like menthol (33–60%), menthone (15–32%), isomenthone (2–8%), 1,8-cineole (5–13%), menthyl acetate (2–11%), menthofuran (1–10%), limonene (1–7%), β-myrcene (0.1–1.7%), β-caryophyllene (2–4%), pulegone (0.5–1.6%), and carvone (1%) have been identified [17]. Freire et al. [36] identified the following main components in Polish peppermint oil: menthol (54.2%), menthone (7.3%), neomenthol (6.3%), carvone (5.0%), and menthyl acetate (4.0%).

On the other hand, thyme oil (Table 3) contained the following main components: thymol (52.91%), *p*-cymene (14.68%), and γ-terpinene (11.15%). Góra and Lis [35] stated that thyme oil is characterized by the presence of thymol (from 16.6% in Italy to 83.2% in Finland), *p*-cymene (from 9.7% in Finland to 36.4% in Poland), and γ-terpinene (from 0.1% in Germany to 12.3% in Italy).

Thyme herb and peppermint leaves contained up to 2.5% and 3.9% essential oil, respectively [39,40].

Peppermint oil possesses antibacterial properties and can be utilized as a natural preservative, extending the shelf life of food for consumption [15]. According to Derwich et al. [41], peppermint oil, due to the presence of monoterpenoid hydrocarbons, exhibits significant antioxidant activity compared to vitamin C, considered a standard antioxidant. On the other hand, the thymol and carvacrol present in thyme oil have been used as natural antioxidants added to sunflower oil [42,43].

**Figure 6:**
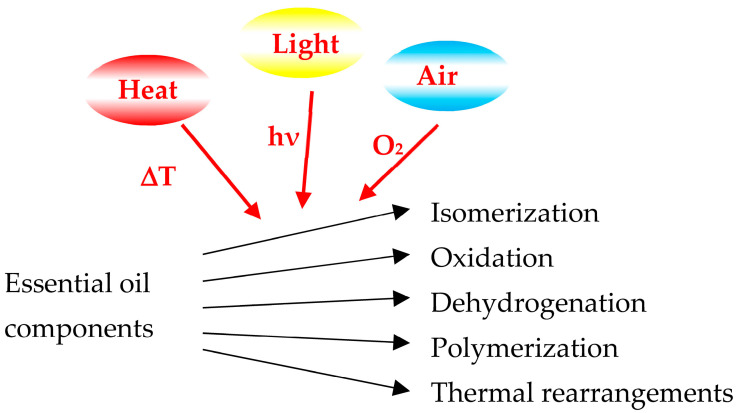
Possible conversion reactions in essential oils [44].

Essential oils are characterized by high instability [44]. They are sensitive to light and prone to oxidation. The occurrence of these processes is indicated by the resinification of essential oils, leading to darkening and thickening. Turek and Stintzing [44] state that temperature significantly influences the stability of essential oil. Generally, heated essential oil may lose stability over extended storage periods and with temperature increases from 0 °C to 28 °C [45], 4 °C to 25 °C [46], and 23 °C to 38 °C [44]. Most cases described in the literature regarding quantitative changes in essential oil components have been induced by exposure to light, temperature, or a combination of both, along with oxygen availability. Therefore, temperature, light, and oxygen availability are considered factors exerting the most significant impact on oil stability [44]. It can be hypothesized that some of the oils used for flavoring fats may contribute to accelerated oxidation. Consideration should also be given to the synergism of the antioxidant activity compounds present in essential oils with the other substances found in sunflower oil. Sicińska [47] points to the cooperation of certain antioxidative substances with other compounds, which may result in changes in the character of the studied compounds, such as accelerated oxidation and other processes altering the profiles of fatty acids, such as cyclization or polymerization (Figure 6) [44]. For these reasons, selecting optimal antioxidant substances for a given fat involves the necessity of conducting further detailed tests for a wide range of concentrations of diverse antioxidants.

Furthermore, one of the reasons contributing to the reduction in the effectiveness of essential oils may be the release of oil components into the surface phase at a temperature of 40 °C. The components of essential oils easily volatilize at temperatures above 35 °C, resulting in a significant decrease in the concentration of these substances in the oil. The impact of essential oil components on the acceleration of quantitative changes in the fatty acid profile could constitute an additional factor resulting in the recording of negative inhibition values. The elevated temperature, in conjunction with UV radiation and the presence of peroxides, may act as a catalyst for the formation of free radicals, thereby initiating adverse reactions among essential oil components. Storage of essential oil at elevated temperatures is not recommended due to its susceptibility to oxidation and polymerization processes influenced by the aforementioned factors. These processes are evidenced by symptoms such as oil transfer to the resin, particularly notable in cases of improper storage [44]. An effective method for prolonging the shelf life of an essential oil involves storing it under refrigeration conditions, effectively mitigating the unfavorable processes outlined above [44]. However, the presence of undesired compounds within the essential oil component profile might also incite reactions leading to unfavorable alterations in the fats.

Despite not demonstrating the effectiveness of adding peppermint and thyme oils in maintaining a stable fatty acid composition in sunflower oil in this study, the search for new natural stabilizers remains relevant as we seek alternatives to synthetic compounds. This need for research is further emphasized by the market availability of various products, including flavored oils. Our model system studies indicate that not all such products exhibit improved properties during storage (stability of fatty acid profile) compared to unmodified oils. Numerous studies suggest that antioxidants can exhibit both antioxidant properties and functions that accelerate oxidation, and that these results are directly linked to the concentration and type of applied antioxidant [6,24]. Currently, due to the popular trend of ecologically beneficial food positively affecting bodily functions, efforts are directed towards reducing the use of synthetic stabilizers. Substantial attention and effort have been devoted to research aimed at finding new sources of antioxidants that perform as effectively as synthetic ones [48]. Natural antioxidants derived from plant extracts are already known, demonstrating a beneficial impact on food quality without harm to human health. Although plant-derived antioxidants are considered harmless to consumers, these additives must meet all safety and health requirements [48].

## 3. Materials and Methods

### 3.1. Experimental Material

The material for the study consisted of the following:

- Sunflower oil (ZT Bodaczów, Poland) purchased at a supermarket in Lublin. The justification for choosing sunflower oil in the experiment was the fact that fats with a significant content of polyunsaturated acids, such as sunflower or soybean oil, are more susceptible to oxidation.
- Peppermint and thyme oils obtained from peppermint leaves (*Mentha × piperita* L.) and common thyme herb (*Thymus vulgaris* L.) produced by Dary Natury (Koryciny, Poland). The essential oil was distilled in accordance with the procedure described in the Polish Pharmacopoeia VIII (2008) [49].

### 3.2. Addition of Antioxidant (BHA) and Peppermint and Thyme Oils

The methodological procedure described in a previous publication was used [6]. In the experimental setup, batches of sunflower oil (50 g in eight replicates for each experimental variant) were meticulously prepared in glass vessels. To these batches, a solution of BHA (butylhydroxyanisole) in ethanol was added at a concentration of 2 mg/cm^3^. Additionally, peppermint and thyme oils were incorporated, resulting in the following concentrations:

- BHA: 0.06%, 0.12%, 0.18%;
- Peppermint and thyme oils: 0.06%, 0.09%, 0.12%, 0.15%, 0.18%, 0.21%.

### 3.3. Storage of Samples and Materials for Analyses

The methodological procedure outlined in a prior publication was employed [6]. Samples of sunflower oil enriched with BHA, peppermint, and thyme oils underwent storage conditions at both room temperature with exposure to daylight and in a thermostat set at 40 °C. The samples subjected to the 40 °C condition were analyzed for fatty acid composition after 2, 4, 6, 8, and 11 months, while those stored at room temperature were analyzed after 11 months. Approximately 50 mg portions were extracted from each of the eight replicates, followed by esterification, saponification, and chromatographic analysis. Initial oil samples, untreated with any reagents (labeled as “0” samples), were similarly collected for analysis.

### 3.4. Determination of Fatty Acids

The methodological procedure described in a previous publication was used [6]. Approximately 50 mg of fat samples were precisely weighed into 20 mL capacity glass ampules. A volume of 0.1 mL of the hexane solution of the internal standard (heptadecanoic acid at a concentration of 10 mg/mL) was introduced to the fat sample [25]. The procedures for fat saponification and fatty acid esterification were conducted following previously established methodologies [26,28,50].

Gas chromatography (GC) was carried out using a Varian GC 450 gas chromatograph equipped with a flame-ionization detector (FID) and a 30 m (0.32 mm i.d.) column coated with a 0.25 µm film of SelectTM Biodiesel for FAME. A temperature gradient was applied (200 °C for 10 min, then incremented by 3 °C/min to 240 °C, holding at 240 °C for 5 min). The injection port and detector temperatures were set at 250 °C and 300 °C, respectively, with a split ratio of 1:50. The flow rates were adjusted to achieve a ratio of gas flows (column + carrier gas):(detector supply):(air) at 1:1:10, with carrier gas (helium) at 28 mL/min, detector supply (hydrogen) at 30 mL/min, and detector supply (synthetic air) at 300 mL/min.

Quantitative analysis relied on calibration curves established for FAMES standard mixture (C14–C22) within the concentration range of 0.1–80.0 g/100 g.

### 3.5. Determination of Essential Oils’ Chemical Composition

The methodological procedure outlined in a prior publication was employed [5].

### GC Analysis

GC/MS

The ITMS Varian 4000 GC-MS/MS system (Varian, Palo Alto, CA, USA), equipped with a CP-8410 auto-injector and a VF-5 ms column (Varian, USA) measuring 30 m × 0.25 mm i.d. with a film thickness of 0.25 μm, was utilized. Helium served as the carrier gas with a flow rate of 0.5 mL/min, while the injector and detector temperatures were set at 250 °C and 200 °C, respectively. The split ratio was maintained at 1:50, and the injection volume was 5 μL. A temperature gradient was implemented (50 °C for 1 min, then incremented by 4 °C/min to 250 °C, followed by a 10 min hold at 250 °C). Ionization energy was set at 70 eV with a mass range of 40–870 Da and a scan time of 0.80 s.

GC/FID

The GC Varian 3800 (Varian, Palo Alto, CA, USA), featuring a CP-8410 auto-injector and a 30 m × 0.25 mm DB-5 column (J&W Scientific, Folsom, CA, USA) with a film thickness of 0.25 μm, was employed. Helium served as the carrier gas with a flow rate of 0.5 mL/min. The injector and detector temperatures were set at 260 °C and the split ratio was maintained at 1:100 with an injection volume of 5 μL. A temperature gradient was implemented (50 °C for 1 min, then incremented by 4 °C/min to 250 °C, followed by a 10 min hold at 250 °C).

The procedure of qualitative and quantitative analysis described in a previous publication was used [6].

The qualitative analysis involved the examination of MS spectra and retention indices [38], along with quantitative analysis utilizing the internal standard addition method (using alkanes C12 and C19). Furthermore, the presentation of essential oil components’ percentages was conducted based on the assumption that the sum of peak areas for all identified constituents equaled 100%.

### 3.6. Determination of Properties of Peppermint and Thyme Oils Inhibiting Changes in Fatty Acid Composition Compared to BHA

The methodological procedure outlined in a prior publication was employed [6,24].

The determination of the activity of the studied peppermint and thyme oils in the experimental system was based on the calculated inhibition Ih, which was derived from the quantitative changes observed in linoleic acid (C18:2) and the total fatty acids (Σ) [6,24].

I_h18:2_ = (C_aox18:2_/C_k18:2_ × 100%) − 100%

where I_h18:2_ is the inhibition of quantitative changes of linoleic acid, C_aox18:2_ is linoleic acid concentration in the sample with essential oil/BHA addition, and C_k18:2_ is linoleic acid concentration in the control sample without essential oil/BHA addition [6,24].

I_hΣ_ = (C_aoxΣ_/C_kΣ_ × 100%) − 100%

where I_hΣ_ is the inhibition of quantitative changes relative to the total amount of fatty acids, C_aoxΣ_ is the total concentration of fatty acids (%) in the sample tested with essential oil/BHA addition, and C_kΣ_ is the total concentration of fatty acids (%) in the control without essential oil/BHA addition. 

### 3.7. Statistical Analysis

The data underwent analysis of variance (Duncan’s test) at a significance level of 5% using the SAS statistical system (SAS Version 9.1, SAS Inst., Cary, NC, USA).

## 4. Conclusions

Quantitative changes in the fatty acid profile of sunflower oil were notably influenced by storage conditions, revealing a significant impact on the oil’s composition over time. This study highlights the detrimental influence of storage conditions on the quantitative aspects of the fatty acid profile, shedding light on a well-established phenomenon in the field. These findings emphasize the importance of understanding the dynamic nature of fat transformations during storage.

Our results further indicate that the inhibitory capacity of BHA against unfavorable fat transformations is intricately linked to both the concentration of BHA in the fat and the storage temperature. This nuanced relationship underscores the need for precise control over these variables when considering the application of synthetic additives like BHA.

Interestingly, the addition of peppermint and thyme oils did not exhibit the anticipated inhibitory effect on changes in the fatty acid composition of sunflower oil. Contrary to expectations, these essential oils influenced a reduction in fatty acids in the examined systems. These unexpected outcomes prompt a deeper exploration into the specific mechanisms at play and the varying effects of essential oils on different edible oils.

In light of previously published results, which demonstrated a positive stabilizing effect on the composition of rapeseed oil with the addition of marjoram oil, there arises a compelling avenue for expanding future studies. Such investigations should encompass a broader range of edible oils and diverse essential oils. The effectiveness of applied additives in the form of essential oils is intricately tied to their chemical composition, the concentration used, and specific storage conditions. These nuanced studies align with current trends in sustainable development and pave the way for the substitution of synthetic compounds with less harmful natural substances, aligning with consumer preferences for healthier and more sustainable options.

## Figures and Tables

**Table 1:** Fatty acid composition of initial sunflower oil.

Fatty Acid	Concentration (g/100 g)
Palmitic acid 16:0	7.40 ± 0.23
Stearic acid 18:0	2.67 ± 0.16
Oleic acid 18:1 cis	22.18 ± 0.84
Linoleic acid 18:2	57.91 ± 0.43
α-Linolenic acid 18:3 α	0.45 ± 0.03
Arachidic acid 20:0	0.20 ± 0.04
*cis*-11-Eicosenoic acid 20:1	0.24 ± 0.04
Σ	91.05 ± 1.05

**Table 2:** Chemical composition of individual components in peppermint essential oil.

Compound	RI	RI_Lit_	Percentage
α-Pinene	938	932	0.52%
Camphene	955	946	tr.
Sabinene	976	969	0.28%
β-Pinene	982	974	0.67%
Myrcene	993	988	0.14%
2-Octanol	1004	994	tr.
*p*-Cymene	1028	1020	tr.
Limonene	1031	1024	0.49%
1,8-Cineole	1034	1026	3.73%
(*Z*)-β-Ocimene	1037	1032	tr.
γ-Terpinene	1060	1054	tr.
*cis*-Sabinene hydrate	1073	1065	0.05%
Linalool	1101	1095	0.17%
3-Octanol acetate	1118	1120	tr.
*cis*-Sabinol	1144	1135	0.07%
*trans*-Sabinol	1146	1137	0.12%
*trans*-Verbenol	1150	1140	0.10%
*neo*-Isopulegol	1152	1144	0.17%
Menthone	1160	1148	31.26%
*iso*-Menthone	1170	1158	8.44%
*neo*-Menthol	1174	1161	2.56%
Menthol	1183	1167	25.71%
*iso*-Menthol	1194	1179	0.48%
*neo*-*iso*-Menthol	1198	1184	0.12%
Pulegone	1249	1233	0.71%
Piperitone	1266	1249	1.18%
*neo*-Menthyl acetate	1279	1271	0.22%
Menthyl acetate	1297	1294	10.50%
*iso*-Menthyl acetate	1314	1304	0.36%
α-Copaene	1382	1374	0.06%
β-Bourbonene	1390	1387	0.47%
β-Elemene	1394	1389	0.61%
*E*-Caryophyllene	1429	1417	3.33%
β-Copaene	1440	1430	0.10%
*cis*-Muurola-3,5-diene	1457	1448	0.12%
(*E*)-β-Farnesene	1461	1454	0.22%
α-Humulene	1469	1452	0.17%
*cis*-Muurola-4(14),5-diene	1475	1465	0.23%
γ-Muurolene	1488	1478	0.06%
Germacrene D	1496	1484	3.38%
β-Selinene	1505	1489	tr.
Bicyclogermacrene	1511	1500	0.30%
Germacrene A	1522	1508	0.12%
γ-Cadinene	1527	1513	tr.
δ-Cadinene	1530	1522	0.14%
*cis*-Calamenene	1534	1528	0.05%
α-Cadinene	1549	1537	tr.
Spathulenol (-)	1588	1577	0.15%
Caryophyllene oxide	1593	1582	0.39%
Viridiflorol	1605	1592	0.47%
1,10-di-epi-Cubenol	1625	1618	0.06%
τ-Muurolol	1658	1642	0.20%

RI—retention indices (from temperature programming, using definition of Van den Dool and Kratz [37]); RI_Lit_—retention indices from literature [38]; tr.—less than 0.05%.

**Table 3:** Chemical composition of individual components in thyme essential oil.

Compound	RI	RI_Lit_	Percentage
Heptane	702	700	tr.
3-metyhyl-3-Buten-1-ol	729	723	0.07%
Hexanal	805	801	tr.
3*E*-Hexenol	854	844	tr.
*N*-Hexanol	867	863	tr.
α-Thujene	927	924	0.92%
α-Pinene	935	932	0.98%
Camphene	952	946	0.42%
Sabinene	974	969	tr.
β-Pinene	982	974	0.48%
1-Octen-3-ol	983	974	0.38%
Myrcene	992	988	2.21%
2-Octanol	1001	994	0.11%
α-Phellandrene	1010	1002	0.25%
δ-3-Carene	1015	1008	2.38%
α-Terpinene	1016	1014	0.10%
*p*-Cymene	1032	1020	14.68%
Limonene	1034	1024	0.32%
β-Phellandrene	1035	1025	0.14%
1,8-Cineole	1037	1026	0.27%
γ-Terpinene	1063	1054	11.15%
*cis*-Sabinene hydrate	1075	1065	0.77%
Terpinolene	1088	1086	0.14%
*p*-Cymenene	1095	1089	tr.
Linalool	1105	1095	2.09%
*trans*-Sabinene hydrate	1108	1065	tr.
Camphor	1153	1141	tr.
Borneol	1179	1165	0.17%
Terpinen-4-ol	1185	1174	0.40%
γ-Terpineol	1213	1199	0.16%
Thymol, methyl ether	1234	1232	0.74%
Carvacrol, methyl ether	1242	1241	0.52%
Geranial	1269	1264	tr.
Thymol	1311	1289	52.91%
Carvacrol	1316	1298	2.34%
Thymol acetate	1351	1349	0.07%
Eugenol	1359	1356	0.07%
α-Copaene	1378	1374	tr.
Isobornyl propanoate	1380	1383	tr.
β-Bourbonene	1386	1387	tr.
Methyl eugenol	1407	1401	0.12%
E-Caryophyllene	1423	1417	1.38%
β-Copaene	1432	1430	tr.
Aromadendrene	1440	1439	tr.
α-Humulene	1457	1452	0.09%
Geranyl propanoate	1473	1476	0.18%
γ-Muurolene	1477	1478	0.12%
Germacrene D	1483	1484	tr.
γ-Amorphene	1494	1495	tr.
α-Muurolene	1500	1500	tr.
β-Bisabolene	1510	1505	0.08%
γ-Cadinene	1516	1513	0.22%
δ-Cadinene	1520	1522	0.27%
α-Cadinene	1539	1537	tr.
α-Calacorene	1544	1544	tr.
Elemicin	1553	1555	0.15%
Geranyl butanoate	1559	1562	tr.
Spathulenol	1580	1577	tr.
Caryophyllene oxide	1585	1582	0.25%
Humulene epoxide II	1613	1608	tr.
1,10-di-epi-Cubenol	1618	1618	0.06%
10-epi-γ-Eudesmol	1626	1622	0.13%
1-epi-Cubenol	1631	1627	tr.
τ-Cadinol	1646	1640	0.42%
α-Cadinol	1659	1652	0.09%
7-epi-α-Eudesmol	1665	1662	tr.
14-hydroxy-9-epi-(*E*)-Caryophyllene	1675	1668	tr.
2*Z*,6*Z*-Farnesol	1698	1698	0.14%

RI—retention indices (from temperature programming, using definition of Van den Dool and Kratz [37]); RI_Lit_—retention indices from literature [38]; tr.—less than 0.05%.

## Data Availability

Data are contained within the article.
